# Research information systems and knowledge graphs: a review

**DOI:** 10.3389/frma.2026.1786866

**Published:** 2026-05-04

**Authors:** Muhammad Haris, Sören Auer, Markus Stocker

**Affiliations:** TIB—Leibniz Information Center for Science and Technology, Leibniz University Hannover, Hannover, Germany

**Keywords:** research information systems, scholarly communication, scholarly knowledge, research data management, knowledge graphs, interoperable information systems

## Abstract

Digital research artifacts (articles, datasets, software, etc.) are the basis for scientific research. Due to the continuous growth and complexity (e.g., due to formats) of such artifacts, ensuring their organization and long-term availability for research is becoming increasingly challenging. It is essential to manage the artifacts so that they are easily discoverable, accessible and useable by relevant communities. Research Information Systems (RISs) have become indispensable in curating, managing, and publishing research artifacts and other research objects. Diverse communities actively use the data from these systems to conduct research-intensive activities across various fields, including computer science, engineering, and life sciences. We review the current state of the art in research information systems, specifically: scholarly identifier systems, bibliographic databases, Research Data Management (RDM) services, and Knowledge Graphs (KGs). These infrastructures play a crucial role in the management of research artifacts. First, we discuss infrastructures that enable the persistent identification of research artifacts to make them globally discoverable and citeable. Second, we discuss databases that manage metadata about research artifacts. Third, we present RDM services that support publishing and accessing research data. Finally, we provide a comprehensive overview of domain-specific and domain-agnostic KGs and databases that have been widely adopted to represent scientific knowledge in different domains in structured form.

## 1 Introduction

Research artifacts (articles, datasets, software, etc.) are proliferating rapidly in various formats on numerous repositories (Hendler, 2014). The traditional scholarly publishing model, which includes peer-reviewed conferences and journals, continues to witness a steady increase in the number of articles (Larsen and von Ins, 2010). Fortunato et al. (2018) suggested that the amount of scientific literature is exponentially increasing, doubling on average every 15 years. While the growth of research artifacts is positive, concerns have been raised about the sheer volume, including of articles and the difficulty for researchers to keep track of the latest developments in their field (Landhuis, 2016). Considering the example of COVID-19, its outbreak led to a global health crisis, researchers from various fields started to investigate different aspects of the virus and its impact on human health. As a result, many artifacts (e.g., articles, datasets) reporting COVID-19 results were published (Guinart and de Filippis, 2020). The large number of articles related to COVID-19 pose challenges for researchers that need to keep track of the latest developments in understanding the virus.

The need for advanced Research Information Systems (RISs) is thus clearly motivated by the need to manage the increasing volume and complexity of research artifacts produced globally by the research community (Fabre et al., 2021; Amorim et al., 2015). Without these systems, research artifacts and the data/information/knowledge therein expressed will unlikely stand the test of time - never be available or become unavailable over time (Surkis and Read, 2015). RISs address this problem by providing the basis for the reliable exchange of scientific knowledge, ensuring that research findings are (easily) accessible, and that the data supporting research findings are preserved and can be reused. Furthermore, given the relevance of interdisciplinary research to modern societal challenges, these systems enable the integration of knowledge across traditional boundaries, making them indispensable tools in the pursuit of a comprehensive understanding of complex problems (Patel, 2016).

**Figure 1:**
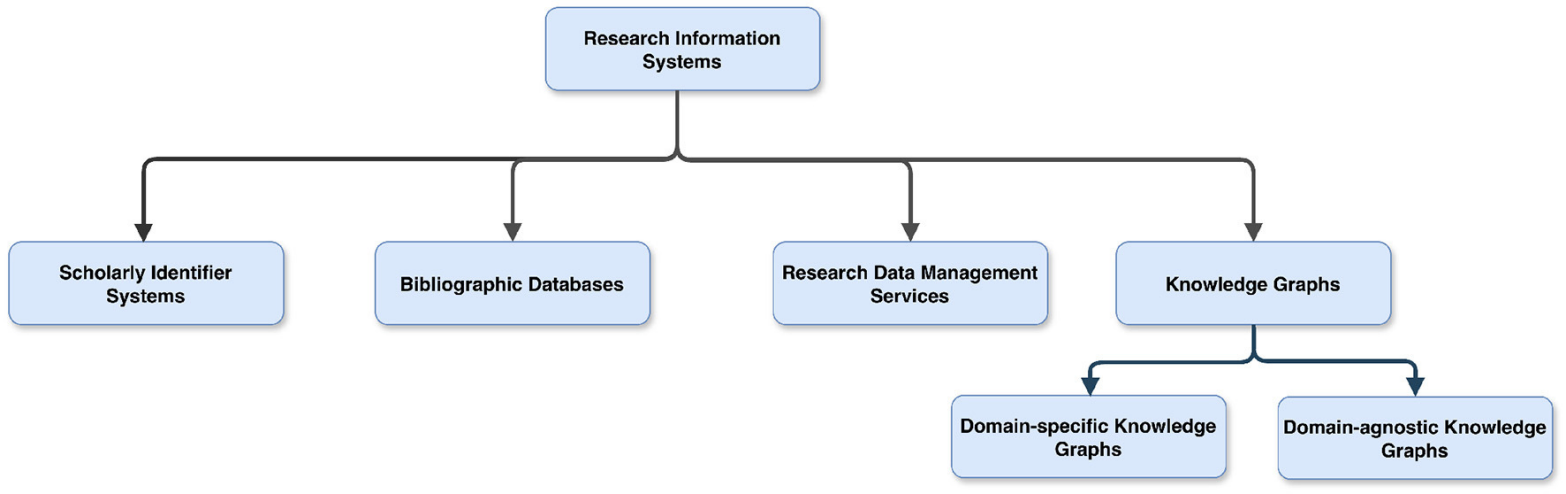
Visual representation of different Research Information Systems, depicting the top node as Research Information Systems, which is further classified into four sub-nodes, namely, Scholarly Identifier Systems, Bibliographic Databases, Research Data Management services, and Knowledge Graphs (KGs). The KGs are further divided into two categories domain-specific and domain-agnostic KGs.

**Figure 2:**
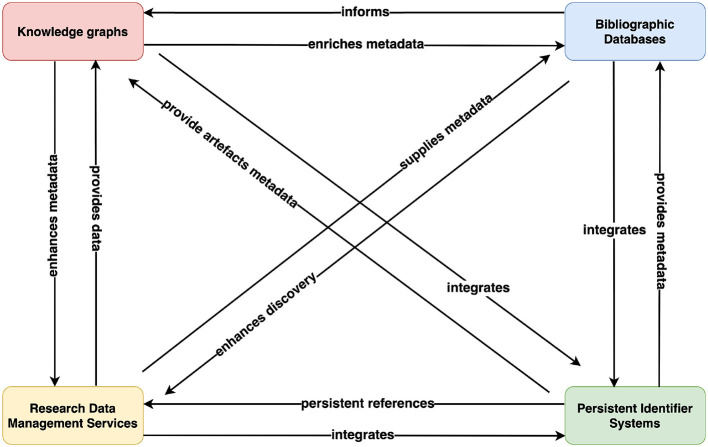
Showing the interaction among Research Information Systems, illustrating how Knowledge Graphs, Research Data Management Services, Bibliographic databases, and Scholarly Identifier Systems integrate and mutually enhance each other's functionalities. Arrows denote the flow of data and highlights the relationships that improve data management, retrievability, and metadata enhancement across different RISs.

Different Research Information Systems—namely, Persistent Identifiers Systems, Research Data Management (RDM) services, and Knowledge Graphs (KGs)—have been developed with the aim of improving the reuse of research artifacts. These systems allow data/information/knowledge to be stored, processed, and disseminated in various formats to meet the diverse information needs of research communities. The primary purpose of these systems is to aid researchers in publishing research artifacts, which increases data discovery, improves reproducibility, facilitates data integration, knowledge synthesis, as well as promotes data citation and attribution. Figure 1 shows the hierarchical view of the different RISs, whereas Figure 2 provides a broader overview of the possible interactions between these systems. In the scholarly communication ecosystem, Persistent Identifier (PID) systems play a fundamental role by assigning unique and persistent identifiers to a wide array of scholarly artifacts, ensuring consistent reference and traceability across research information systems. These identifiers are crucial for linking information within Research Data Management (RDM) services that manage the storage, preservation and accessibility of research data. RDM services utilize PIDs to make datasets and other research outputs persistently identifiable and citeable, and they export the metadata of research artifacts to Bibliographic databases. Bibliographic databases leverage these identifiers to accurately catalog and link artifacts, thereby creating a searchable database that enhances the discoverability of these research artifacts. In addition, these unstructured or semi-structured data and metadata are integrated into knowledge graphs to build detailed, interconnected networks of research artifacts. By using PIDs for precise linking, the content of knowledge graphs can be linked in a meaningful way to different research results. Together, these systems form a coherent framework that supports the entire lifecycle of scholarly communication and enhances the integrity, accessibility and utility of research outputs across various disciplines. For a detailed discussion of how these systems interact and link scientific artifacts, see Section 7.

We present the current state of these RISs and highlight their key features that contribute to sharing, publishing and reusing research artifacts. We start by introducing and comparing Scholarly Identifier Systems that serve as the foundation for building scholarly communication infrastructures. Second, we provide an overview of various RDM services that are crucial for publishing research artifacts (articles, datasets, scientific reports, and other objects) across different domains. Third, we discuss Bibliographic databases that provide rich metadata information about several research artifacts. Fourth, we present domain-specific knowledge graphs that organize information for specific research areas, followed by domain-agnostic knowledge graphs. In addition, we discuss the shortcomings identified in the analysis of these systems, as well as various future directions to further improve the production, sharing, and accessibility of these research artifacts.

Our contributions are as follows:

- We provide a comprehensive overview of existing Research Information Systems, specifically scholarly identifier systems, bibliographic databases, research data management services, and knowledge graphs. This overview highlights their functionalities and significance in sharing, publishing, and reusing research artifacts.
- We analyze the interrelationships between these systems and how they collectively support the ecosystem of scholarly communication. Rather than treating these components in isolation, the review synthesizes their roles and interactions within contemporary scholarly communication infrastructures, thus demonstrating how their integration enables dynamic information flow and improves the discovery, accessibility, and citation of research artifacts.
- We further emphasize the resources that are crucial for conducting the research. This includes a description of the datasets used, which provides information on the scope and reliability of the research findings. We also outline the software implemented in these studies and highlight specific tools or algorithms that were important for knowledge extraction. With this information, we aim to provide a comprehensive overview of the framework and tools underpinning the research, thus allowing readers to fully understand the methodologies and results presented.

To further enrich this review, we create structured descriptions of the reviewed papers using the Open Research Knowledge Graph (ORKG) (Auer et al., 2020; Stocker et al., 2023). The ORKG is a production research infrastructure designed to represent scientific knowledge in a machine actionable, structured and semantic manner and provides services to compare knowledge, in tabular and other visual forms, among other kinds of services for efficient scientific knowledge reuse. ORKG enables the structured description of research contributions published in articles, whereby a contribution represents the addressed research problem, the employed materials and methods, and an obtained result. In addition, the ORKG allows comparison (Oelen et al., 2020b) of scientific knowledge and thus supports knowledge synthesis.

Numerous state of the art reviews related to knowledge graphs (Ji et al., 2022; Tian et al., 2022; Abu-Salih, 2021) have been published that offer comprehensive summaries of the research articles. However, these reviews are primarily document-centric and lack structured descriptions. This deficiency leads to difficulties in accessing vital content for further use. Since scholarly articles contain important scientific information, ensuring compliance with the Findable, Accessible, Interoperable, and Reusable (FAIR) principles (Wilkinson et al., 2016) is essential for enabling advanced applications. By leveraging the ORKG machine-readable descriptions, we can conduct data-driven analysis, create visualizations, and address a wide array of questions, particularly those related to quantitative analysis, for example:

1. How frequently is a specific method employed in text processing to generate knowledge graphs?
2. Which data storage structure is adopted to store the knowledge graph data?
3. Identify the research papers that offer data and software implementations for the creation of knowledge graphs.
4. Identify papers that have reported the evaluation of end results.

By describing the essential information of articles in the ORKG, the aforementioned questions can be easily answered, which will simplify the search of relevant research articles. This approach allows users to tailor their exploration of the literature, focusing on articles that meet specific criteria.

### 1.1 Methodology for literature search

This study adopts a systematic and structured literature search methodology to identify and analyze relevant research on Research Information Systems (RIS), scholarly infrastructures, and knowledge graphs. The methodology follows three main steps: (i) defining the research problem, (ii) identifying relevant literature through a keyword-based search strategy, and (iii) extracting and analyzing relevant information from the selected publications.

#### 1.1.1 Search method

An iterative keyword-based search was conducted across five major scholarly databases: IEEE Xplore, SpringerLink, ScienceDirect, Frontiers, and the ACM Digital Library. These databases were selected to ensure broad coverage of computer science, information systems, and interdisciplinary research related to scholarly infrastructures and knowledge graphs. The review includes peer-reviewed journal articles and conference proceedings indexed in these databases.

A detailed set of keywords was defined to capture different facets of Research Information Systems, scholarly identifier systems, bibliographic databases, research data management platforms, and knowledge graphs. Keywords were applied both individually and in combination using Boolean operators such as AND and OR to refine the search results. For example, combinations such as “Persistent identifiers” AND “Scholarly identifiers” were used to retrieve publications explicitly addressing both concepts. Quotation marks were applied to enforce exact phrase matching where appropriate. Table 1 summarizes the keywords used in the search process.

**Table 1:** Keywords used to search papers related to different research information systems.

System	Keywords	Search strategy
Scholarly identifier Systems	Persistent identifiers; scholarly identifiers; persistent identifiers for research artifacts; organizations for persistent identifiers	Keywords searched individually and in combinations using AND/OR. Example: “Persistent identifiers” AND “Scholarly identifiers”
Research data management	Research data management platforms; research data management services; scientific data management platforms; scientific data management services; research data management repositories	Each set of terms is searched separately.
Bibliographic databases	Bibliographic databases; scholarly communication infrastructures; citation databases	Each set of terms is searched separately.
Knowledge graphs (domain-specific and domain-agnostic)	Life sciences knowledge graphs; healthcare knowledge graphs; engineering knowledge graphs; food sciences knowledge graphs; mathematics knowledge graphs	Each set of terms searched separately; all terms other than “domain-specific knowledge graphs” and “domain-agnostic knowledge graphs” were searched within double quotation marks.

#### 1.1.2 Types of contributions covered

The literature included in this review spans two complementary perspectives. First, we consider *model-centric* contributions that primarily focus on conceptual models, architectures, methods, or empirical studies related to knowledge graphs and research infrastructures. Second, we analyze *system-centric* contributions that describe the design, implementation, and deployment of concrete platforms, services, or infrastructures, such as research management platforms, bibliographic databases, and research data management systems. By jointly considering both perspectives, this review captures methodological and conceptual advances as well as their realization in operational scholarly systems.

#### 1.1.3 Screening procedure

The initial keyword-based search returned several hundred publications, which were then filtered through a multi-stage screening process to obtain a thematically focused corpus. First, titles and abstracts were screened to remove clearly irrelevant or off-topic publications. Subsequently, full-text screening was conducted for the remaining papers to assess their relevance and depth with respect to the objectives of this review. This process ensured that only publications providing substantial conceptual, methodological, or empirical contributions were retained for analysis.

#### 1.1.4 Inclusion and exclusion criteria

To ensure relevance and analytical consistency, explicit inclusion and exclusion criteria were applied throughout the screening process. Publications were included if they (i) addressed Research Information Systems, scholarly infrastructures, or knowledge graphs in academic or research contexts; (ii) described concrete systems, platforms, architectures, tools, or frameworks; and (iii) provided conceptual, methodological, or empirical insights relevant to scholarly communication or research data management.

Publications were excluded if they (i) focused on non-scholarly application domains such as tourism, politics, or commercial services; (ii) mentioned RIS-related concepts only marginally without substantive discussion; or (iii) lacked sufficient technical or conceptual depth relevant to the objectives of this review.

After applying the screening procedure and selection criteria, a total of 69 publications were retained for detailed analysis. While it is not feasible to include every relevant publication in a single review, the final selection prioritizes thematically aligned contributions that provide conceptual depth, system-level descriptions, or empirical insights.

### 1.2 Scope of analysis and selection rationale

#### 1.2.1 Selection of bibliographic databases and RDM platforms

Beyond the literature search, this review examines a set of representative bibliographic databases and research data management (RDM) platforms that manage the (meta)data of research artifacts. The selection of these platforms was guided by their alignment with the FAIR data principles, which constitute a widely accepted framework for improving the findability, accessibility, interoperability, and reusability of research artifacts. Each platform was analyzed with respect to criteria such as the availability of rich metadata, the use of persistent identifiers, support for programmatic access via APIs, and interoperability mechanisms. The selected platforms represent both domain-agnostic and domain-specific solutions and play a significant role in contemporary scholarly communication.

#### 1.2.2 Selection of domain-specific knowledge graphs

In addition, this review considers domain-specific knowledge graphs developed across multiple research areas, including education, healthcare, and computer science. These domains were selected to demonstrate the broad applicability of knowledge graphs in addressing domain-specific scholarly challenges and supporting research and academic workflows. Domains unrelated to scholarly or academic activities, such as travel or politics, were excluded to maintain a clear focus on research-oriented use cases. The selected domains illustrate how knowledge graphs can be adapted to heterogeneous data sources, disciplinary practices, and user needs within scientific and academic contexts.

## 2 Background

This section introduces the concepts relevant to the work. Additionally, we compare the Open Research Knowledge Graph (ORKG) with other publicly available knowledge graphs and provide a rationale for choosing ORKG as a supporting tool. This comparison aims to highlight the unique features and advantages of ORKG that make it particularly suitable for our purposes.

### 2.1 Research information systems

According to Laudon and Laudon (2004), an information system can be defined as a *set of interrelated components that collect, process, store, and distribute information to support decision making and control in an organization*. It typically includes data on publications, projects, funding sources, patents, and other aspects of scholarly work. RIS is a broader or more generic term for any system used to manage research-related information and developed as platforms that integrate various research data components. Essentially, RIS serves as an umbrella category encompassing a broad range of systems used for managing research-related information across academic and research institutions.

### 2.2 Bibliographic databases

Bibliographic databases are widely recognized as digital repositories that contain references to published materials, particularly journal articles and conference proceedings. These references are cataloged with specific details such as titles, author names, affiliations, and abstracts (Gasparyan et al., 2013).

### 2.3 Research data management service

Research Data Management service is a specialized support service that assists researchers in organizing, storing, managing, preserving, and sharing research artifacts across different domains (Patel, 2016), thereby enhancing the utility and impact of the research data.

### 2.4 Knowledge graphs

A knowledge graph is an advanced data structure that consists of interconnected entities (such as objects, events, situations, or concepts) represented as nodes, and the relationships between these entities represented as edges (Hogan et al., 2022). This graph-based model enables data to be stored in a way that emphasizes the interconnectivity of concepts across different domains.

### 2.5 Rationale of using ORKG

The ORKG is designed specifically to present the essential information of articles in structured and comparable form. Scientific knowledge published by ORKG is Findable, Accessible, Interoperable, and Reusable (FAIR) (Wilkinson et al., 2016). It makes the scientific knowledge interlinked and understandable to machines. While this article provides concise summaries of the reviewed papers, we create detailed and structured descriptions of these papers as ORKG Papers and Comparisons. There exist a number of publicly available knowledge graphs [specifically, SemOpenAlex (Färber et al., 2023), Computer Science Knowledge Graph (Desśı et al., 2022), and SoftwareKG (Schindler et al., 2020)] that present the (bibliographic) metadata of articles in a structured manner.

SemOpenAlex and Software KG primarily focus on metadata that typically includes metadata about various entities and their relationships, such as titles, authors, publication dates, affiliations, and references, whereas the Computer Science Knowledge Graph (CS-KG) is a large-scale automatically generated knowledge graph describing millions of statements extracted from CS articles about 24M entities (e.g., tasks, methods, materials, and metrics).

Unlike these knowledge graphs, ORKG is designed to capture and represent scientific knowledge in a structured format. This includes not just metadata but the actual content of articles. Furthermore, in contrast to other KGs, the ORKG provides services that enable the production of structured descriptions as well as the comparison of contributions. These rich functionalities motivated the use of the ORKG to structure and organize the work presented in our manuscript.

## 3 Scholarly identifier systems

This section provides a system-centric synthesis of scholarly identifier systems that are foundational to research information systems. Rather than aiming for an exhaustive review of all publications in this area, we focus on widely adopted identifier infrastructures that are most frequently discussed in the literature and play a central role in scholarly communication.

The basis for research information systems are scholarly identifier systems. Scholarly identifier systems are designed to provide unique and persistent identifiers for different entities including research artifacts and other objects. These entities can include authors, institutions, datasets, research articles, and other scholarly works. The purpose of these systems is to facilitate the identification, disambiguation, and efficient management of aforementioned entities. Key identifiers for scholarly communication include:

- Document Object Identifiers (DOI) provided by DataCite (Rueda et al., 2017) and Crossref.
- ORCID identifiers for researchers (Haak et al., 2012).
- ROR identifiers for research organizations (Gould, 2019).

***Digital Object Identifier (DOI)*** is a widely used identifier for research artifacts such as articles, books, datasets, and samples (Paskin, 2010). DOIs are assigned by publishers and designed to be persistent, i.e., they remain the same even if the location of the object changes. It ensures that the published digital resources have a stable and unique reference. A DOI is often included in citations, providing a direct link to the content's digital location. By offering a permanent address to digital content, the DOI system ensures that even if the URL changes, the content remains easily accessible.

***Researcher Identifier (ORCID)*** enables the unique and persistent identification of researchers (Haak et al., 2012). It aims to solve the problem of authorship ambiguity, allowing researchers to maintain a record of their scholarly work and easily attribute their scholarly contributions.

***Research Organization Registry (ROR)*** provides a unique identifier for research organization, facilitating the disambiguation of organizations and linking them to their research outputs. ROR allows tracking of research outputs of organizations, which is important for funding agencies, academic institutions, and other stakeholders.

Since persistent identifiers have associated metadata, information about artifacts exists independently of the identified artifact. This metadata layer is typically standardized and supports the findability and accessibility of artifacts as well as enables opportunities for metadata linking and sharing among scholarly infrastructures (Meadows et al., 2019). Persistent identification is applied to different research artifacts [datasets (Richards et al., 2011; Haris et al., 2021, 2022a) and software (Jones et al., 2016)] and also adapted for numerous entity types, in particular, instruments (Stocker et al., 2020); geometric and topological entities (Farjana et al., 2016), conferences (Franken et al., 2022), and resources (Haun and Nürnberger, 2013). PIDs are essential in linking research artifacts (papers, datasets and software, and other research data) in scholarly communication infrastructures and such linking further enables the easy discovery of these artifacts. Table 2 provides an overview of articles discussing the persistent identification of different research artifacts and other objects, whereas Table 3 highlights different systems that allow to persistently identify these research artifacts.

**Table 2:** Summary of papers discussing the persistent identification of different entities.

References	Description	Schema
Jones et al. (2016)	Persistent identification of software	DataCite schema
Richards et al. (2011)	Persistent identification of datasets	Biodiversity Vocabularies and Schemas
Stocker et al. (2020)	Providing persistent and unique identifiers for instruments	PIDINST schema
Franken et al. (2022)	Persistent identification of conferences and workshops	Specialized schema for conferences
Farjana et al. (2016)	Persistent identification of topological entities in CAD modeling systems to ensure the persistence of design modifications	Specialized schema for topological entities in CAD models
Haun and Nürnberger (2013)	Persistent identification of information management resources (files, data, or digital assets)	Uniform Resource Identifiers (URIs), Uniform Resource Names (URN) and Locators (URL)

**Table 3:** Comparison of attributes across different identifier systems.

Attribute	DataCite	ORCID	ROR	PID INST
Entity type	Scholarly artifacts	Researchers	Organizations	Instruments
Schema	DataCite metadata schema^a^	ORCID schema^b^	Metadata about an organization including name, alternate names, and location	PID INST schema^c^
Provided services	API (GraphQL, REST), search, publish metadata	API, search	API, entity retrieval, reconciliation, search	API, search
Uses identifiers	DOI	ORCID Identifiers	ROR Identifiers	DOI
Goal	Persistent Identification	Persistent Identification	Persistent Identification	Persistent Identification
Used by	Scholarly infrastructures	Scholarly infrastructures	Scholarly infrastructures and organizations	Scholarly infrastructures

^a^ https://schema.datacite.org/

^b^ https://github.com/ORCID/orcid-model/tree/master/src/main/resources/record_3.0

^c^ https://github.com/rdawg-pidinst/schema/blob/master/schema.rst

The importance of persistent identification of artifacts used in research is discussed by the FAIR data principles (Wilkinson et al., 2016). Persistent identifiers play a crucial role in making research data Findable, Accessible, Interoperable, and Reusable. PIDs make it easier for researchers to locate and access research artifacts. Additionally, PIDs facilitate the linking of data across different systems and repositories, thereby enabling the interoperability of research data. As a result, making research outputs easily discoverable for reuse.

## 4 Bibliographic databases

This section presents a system-oriented overview of major bibliographic databases that support the aggregation, discovery, and interlinking of scholarly metadata. The selection emphasizes infrastructures that are prominently referenced in the literature and that provide programmatic access or graph-based representations of scholarly artifacts.

Bibliographic databases play a critical role in the sharing of research outputs within the scholarly community. There exist several databases that provide information about various scholarly artifacts. Next, we explain different bibliographic databases that have been primarily utilized for managing bibliographic information of different research artifacts.

***DataCite***^1^ provides services for the persistent identification of research artifacts, particularly datasets and software by following the standard metadata schema. Various data sources integrate DataCite services to facilitate users to publish their datasets, software, and supplementary material for their discovery in other research information systems. DataCite also provides the Persistent Identification (PID) Graph (Cousijn et al., 2021; Fenner and Aryani, 2019), which enables federated retrieval of the metadata of numerous research artifacts, specifically articles, datasets, software, and other entities, including organizations, projects, and funders, at large-scale available in various scholarly communication infrastructures. Since these research artifacts are largely associated with persistent identifiers, the relationships among these artifacts are also discoverable in the PID Graph. The PID Graph is accessible via the DataCite GraphQL API.^2^ The integrated and federated access to research artifacts via PID Graph enables addressing complex use cases.

***OpenAIRE*** (Manghi et al., 2012a,b) Research Graph enables the discovery of research artifacts, including research articles, datasets, software, projects, and other entities such as researcher profiles, organizations, and funders. The metadata available in OpenAIRE is sourced from a variety of data providers, curated by domain experts as well as extracted from research articles using various natural language processing methods. To ensure the data uniqueness, different deduplication methods are applied, effectively removing redundancy. The curation process also ensures the linking between articles, datasets, and software, and other entities to make them easily discoverable and accessible. The data available in OpenAIRE is accessible using the REST API and SPARQL endpoint.

***SemanticScholar***: An AI-based search engine—SemanticScholar^3^ enables searching scholarly articles and researcher profiles at large-scale. It employs machine learning and natural language processing methods to improve the publication searching. Its rich REST API allows persistent identifier based and keyword-based queries for searching scholarly articles and researcher profiles.

***Wikidata***^4^ (Nielsen et al., 2017) is a collaborative and multilingual knowledge graph hosted by the Wikimedia Foundation that enables searching research articles and information about other entities (e.g., organization, people, etc.). Data available in Wikidata is accessible via REST API and SPARQL endpoint.

***SciGraph*** is a web tool, an initiative of Springer Nature and based on the concepts of Linked Open Data (LOD). It contains information about scientific research, including publications, research data, affiliations, grants, and patents. SciGraph comprises a diverse range of domains, including computer science, medicine, life sciences, chemistry, engineering, and astronomy, among others. With the implementation of SciGraph, Springer Nature strengthens its role in producing connected research so that researchers can discover research outcomes easily.

***Unpaywall***^5^ is a database of open access articles which harvests the articles metadata from legal sources including repositories and publishers. Several organizations have integrated Unpaywall considering the advantage of providing information of open access content. The served metadata includes the URL to open access version of articles which is accessible via REST API.

***OpenCitations***^6^ (Peroni and Shotton, 2020) is an infrastructure that publishes the bibliographic and citation data using the Linked Data technologies. This infrastructure complies with FAIR principles and is useful for researchers, publishers and other stakeholders to support different research activities. The data stored in OpenCitations is accessible using a REST API and SPARQL endpoint.

### 4.1 Overview of existing services

A series of services have been developed by sourcing the metadata from OpenAIRE. A notable example is the Scholix framework (Burton et al., 2017), in which the links between publications and underpinned datasets as well as other datasets are established, ultimately capturing and exposing rich links between artifacts and making such information globally visible and trackable. OpenAIRE Connect leverages Zenodo to make datasets FAIR and connect them with publications. IntelComp's^7^ STI Data Space was also developed using the structured metadata of OpenAIRE. It is composed of interconnected currently fragmented and dispersed data from various ecosystems. OiPub^8^ is a scientific research discovery and discussion platform built using the research publications retrieved from OpenAIRE. Another service Linknovate^9^ was developed using the organizations information sourced from OpenAIRE. It provides information about different kinds of organizations and latest news about latest trends in different sectors. A federated GraphQL service was developed to retrieve the contextual information about research artifacts from various information systems, where the related projects information was fetched from the OpenAIRE using its API.

DataCite provides a user interface, DataCite Commons^10^—a web-based user interface to access the content served by the PID Graph. A dynamic user interface—Scholia which was developed to access data from Wikidata using its SPARQL endpoint—allows users to search articles, organizations, publishers and researcher profiles. Furthermore, Scholia also aggregates the retrieved information to provide summarized and visualized views to the users, for example, co-citation analysis, research areas of a researcher etc. Similarly, WikiCite^11^ is also a web tool which organizes and allows searching bibliographic information sourced from Wikidata.

## 5 Research data management services

This section provides an overview of existing services that can be used for data management purposes. Research Data Management (RDM) services support the collection, organization, sharing, and preservation of research data (Amorim et al., 2017). In addition, these services play a critical role in facilitating the management and reuse of research data, ensuring that valuable research outputs are persistently stored, discoverable, and accessible for future use.

***Zenodo***^12^ is a general open repository created by the European OpenAIRE program. It facilitates the deposit of research papers, datasets, software, reports, and other digital artifacts relevant to scientific work. Each submission gets a permanent digital object identification (DOI), which facilitates easy citation of the published objects.

***PANGAEA***^13^ stands for Publishing Network for Geoscientific & Environmental Data is a renowned data publisher dedicated to archiving, publishing, and disseminating georeferenced data derived from earth systems research. Managed by the Alfred Wegener Institute and the MARUM Center for Marine Environmental Sciences, this platform serves a wide array of topics including oceanography, geology, climatology, and archaeology. It assigns DOIs to datasets, ensuring they are citable and discoverable. Datasets published with PANGAEA are linked to their respective articles, hence, research material underpinning the research work can also be discovered by following the existing metadata-based relations. PANGAEA and Elsevier collaborates to follow the practice of linking data with publications. The data published on PANGEA is also visible on the landing pages of Elsevier publications, if the publication refers to its supplementary data.

***Figshare***^14^ is an open-access repository allows researchers to share and access scholarly content, including figures, datasets, software, and reports. The content uploaded on figshare is assigned a DOI, ensuring its discovery and citability. It is designed to facilitate open science and the public accessibility of research data, ultimately promoting the sharing of data across all research fields. It serves as a valuable tool for researchers looking to maximize the visibility and impact of their research findings.

***Dryad***^15^ is an open-access repository for publishing research data, mainly in the fields of evolutionary, genetic, and ecological biology. Data available in Dryad is discoverable, freely reusable, and citable. The objective of Dryad is to provide an infrastructure for the reuse of scholarly research data. Dryad aims to enhance the sharing of research data where professional associations, publishers, research and educational institutions, funding agencies, and other stakeholders collaborate to support the preservation and reuse of medical data.

***Harvard Dataverse***^16^ is a global repository to publish scholarly data from all disciplines. It contains the largest collection of social science research data. It hosts data for organizations, projects, archives, and journals.

***Mendeley Data***^17^ is a cloud-based data management repository that allows researchers to store, share, and manage their research data specifically, medical data.

***Vivli***^18^ is a data sharing platform for clinical studies to serve international research communities. It acts like a broker between data contributor, data user, and the data sharing communities. It allows users to retrieve related studies, request data from data contributors, or share data.

***CKAN***^19^ (Comprehensive Knowledge Archive Network) is a data catalog service popular among government organizations for sharing open data. It supports a wide range of file types and provides users with access to both the underlying data and its metadata in a variety of ways, including through visual representations and REST API.

***Atlas of Living Australia***^20^ is an open infrastructure that harvests Australian biodiversity data from multiple data sources, and makes it accessible and reusable for researchers. It helps to create a more detailed landscape of Australia's biodiversity for scientists, policy makers, environmental planners and land managers, industry and the general public, as well as enables them to work more efficiently.

***EarthChem***^21^ offers open data services to the geochemical, petrological, mineralogical, and associated communities, including data preservation, discovery, access, and visualization.

### 5.1 Overview of existing services

PANGAEA implements an interoperability framework based on internationally accepted standards and web-service–based interfaces, enabling broad dissemination of metadata and datasets. This facilitates integration with major discovery services, library catalogs, and scientific data portals, ensuring high findability of hosted data. As a result, PANGAEA supports data access through infrastructures such as GEOSS and GBIF.^22^ This foundational interoperability allows core RDM services to enable advanced services. For instance, Zenodo leverages its repository backbone to support European Commission funding mandates and host community-led collections, while DataCite provides the essential persistent identifier and citation infrastructure that enables tracking and attribution across these distributed services. Commercial platforms like Figshare have built upon this model to offer tailored data hosting and supplementary material management for publishers. These repositories, including Dryad, Figshare, and Zenodo, participate in broader scholarly ecosystems by feeding data-literature link information to hubs like DataCite, which is then aggregated by services such as Scholexplorer to create a global map of research connections (Khan et al., 2020). Indeed, Brinckman et al. (2019) describe how such advanced services leverage core RDM services to create integrated, executable research environments that go beyond static archives.

## 6 Knowledge graphs

In addition to the aforementioned infrastructures, there exist several other scholarly infrastructures, known as knowledge graphs which are structured representations of real-world information. These graphs serve as complex maps of interconnected data and enable a structured and semantic understanding of the information available on the web. In these graphs, real-world entities and their intricate relationships are mapped as nodes and edges. Such nodes and edges represent semantic annotations that enable machines to understand and interpret the represented knowledge (Hogan et al., 2022). These graphs are constructed usually by gathering information from a variety of data sources, including other research graphs. The flexible structure of knowledge graphs makes them adaptable for evolving information and growing knowledge domains. Knowledge graphs follow a holistic paradigm of knowledge organization, where unstructured, semi-structured, and taxonomic information can be represented and managed in a structured way. This has resulted in an increasing interest in knowledge graphs from the industrial side and enabled a vast amount of research w.r.t. knowledge graph reasoning, retrieval, completion, and question answering (Zou, 2020). Knowledge graphs are usually stored in graph databases or dedicated triple stores. Since knowledge graphs can be derived from multiple sources, their structure and details can vary significantly. Such variability is addressed by concepts like ontologies (McGuinness and Van Harmelen, 2004) and taxonomies. The ontology offers a formal description of knowledge as a set of concepts within a particular domain and the relationships that hold between them.

**Figure 3:**
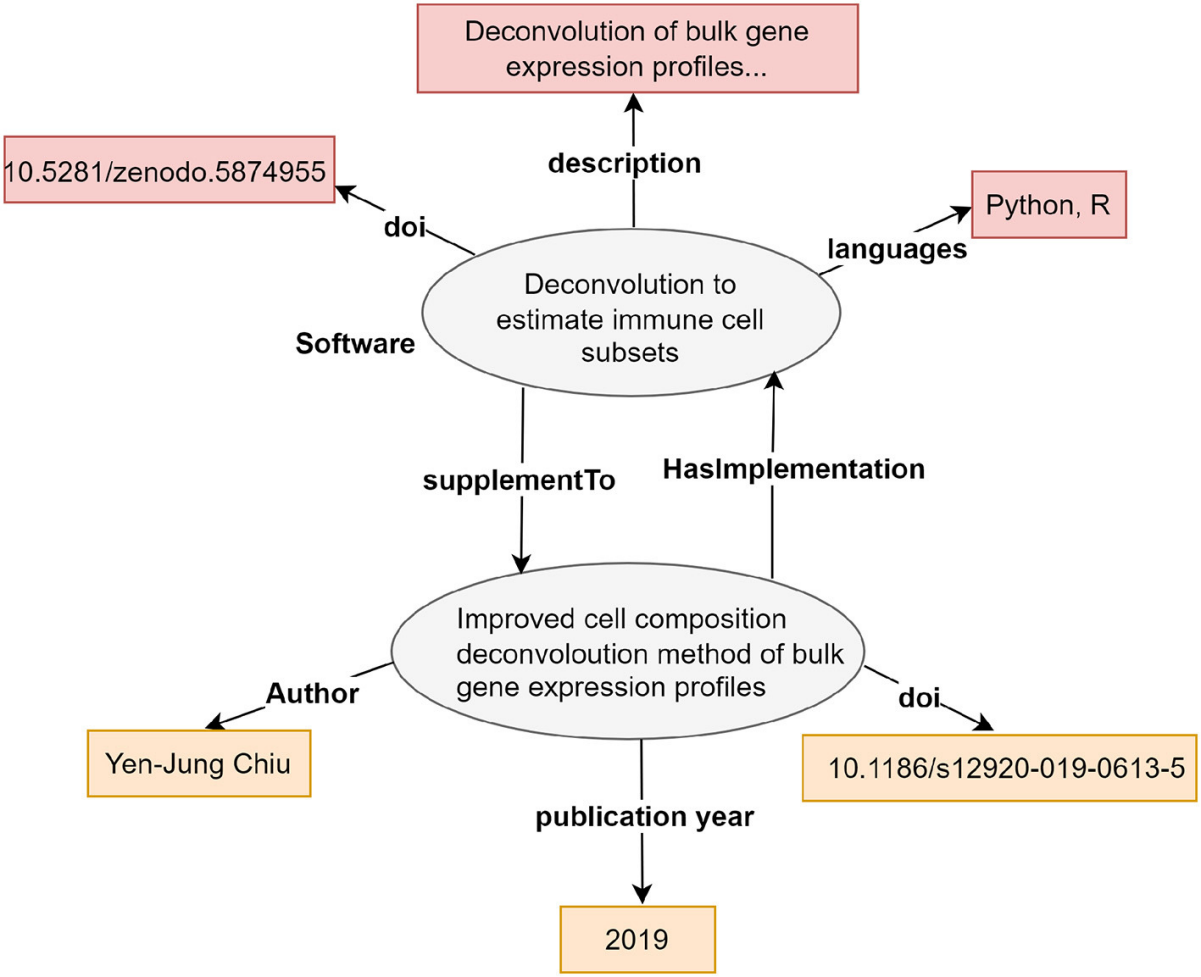
An example of a knowledge graph that illustrates the relationship between an article and its corresponding software implementation. The graph displays the article's title, and links to other entities such as DOI, publication year, and authors, which are linked with their respective properties. Similarly, the software entity is linked with other entities (DOI, languages, and description) using the respective properties.

Information in knowledge graphs is organized in the form of statements, where each statement is expressed as “subject (*s*)—predicate (*p*)—object (*o*),” as shown in Figure 3. These triples form connections between different objects through a combination of resources, properties, and their values. The construction of the knowledge graphs is based on the Resource Description Framework (RDF)^23^ graph structure. In RDF graph, each triple constitutes a statement about a relationship *p* linking the things signified by *s* and *o*. The identifiers for *p, s*, and *o* are URIs (Uniform Resource Identifiers), enabling links in one knowledge graph to reference elements in another knowledge base located elsewhere. In addition to a URI, the object in a triple can also be a literal, such as a string or any XML datatype. While objects identified by URIs can also serve as subjects in other triples, literals cannot act as subjects in further triples.

### 6.1 Domain-specific knowledge graphs

Domain-specific knowledge graphs represent and store information related to specific domains such as healthcare, computer science and engineering. They encapsulate entities, relationships, and concepts that are unique to that domain, offering a structured semantic framework that allows for information retrieval, decision support, and data analysis. These specialized knowledge graphs are instrumental in addressing complex user queries, predicting trends, and providing recommendations that are highly relevant to domain-specific problems. We cover a broad range of domain-specific knowledge graphs, including applications in healthcare, food systems, code and software analysis, cybersecurity, education, geoscience, engineering, and research datasets. For each domain, we summarize the corresponding knowledge graph initiatives and also provide structured tabular overviews that capture the addressed research problems, the methods employed, the data sources used, and the availability of resources. By doing so, the review goes beyond a descriptive overview and highlights aspects related to the reuse and reproducibility of the discussed knowledge graphs and their underlying approaches.

#### 6.1.1 Healthcare knowledge graphs

Healthcare data is rapidly increasing following the characteristics (volume, variety, and velocity) of big data (Dash et al., 2019; Pastorino et al., 2019). The rapid increase in data volume necessitates its representation in a form that machines can understand. Given the importance of healthcare data many KGs have been developed.

Yu et al. (2017) constructed a large-scale knowledge graph of Traditional Chinese Medicine (TCM) health preservation by integrating numerous data resources. This knowledge graph facilitates efficient data retrieval, visualization, and recommendation, thus expediting the utilization of health care knowledge. Gyrard et al. (2018) proposed a personalized health care knowledge graph (PHKG), that incorporates patient's health condition (personalized knowledge) as well as contextual information (diseases symptoms and treatment). Such diversified data is taken from different sources such as Web of data and environmental sensors. The aggregated knowledge in PHKG facilitates physicians in understanding the disease symptoms and treatment plan. Another KG (Malik et al., 2020) was proposed by integrating structured and unstructured data of patients diagnosed with cerebral aneurysms. The KG was enriched with a set of concepts, hierarchical and non-hierarchical relationships, and predictive rules for prediction of subarachnoid hemorrhage, thus helps physicians in clinical decision making. Postiglione (Postiglione, 2021) developed an Italian Healthcare Knowledge Graph by integrating numerous Electronic Health Records (EHRs). The KG allows physicians to interactively identify and explore relations between healthcare entities (symptoms and diseases). Similarly, another healthcare KG was developed by integrating Textual Medical Knowledge (TMK) which supports semantic querying and reasoning (Shi et al., 2017). Huang et al. (2017) proposed disease-centric knowledge graph of depression which integrates numerous knowledge resources (clinical trials, medical publications, and reports). The developed KG helps psychiatric doctors in efficiently exploring and finding answers related to depression. Li et al. (2020) proposed a systematic approach to construct the medical KG which integrates Electronic Medical Records (EMRs) and provides basis for many medical related applications. KnowLife (Ernst et al., 2014), a large-scale knowledge base for health and life sciences constructed by retrieving data from multiple web sources. KnowLife is an extensive KG that contains a variety of relations about diseases, symptoms, causes, risk factors, drugs, side effects, and many other factors. Several other healthcare knowledge graphs (Zhang et al., 2020; Yu et al., 2022; Huang et al., 2019; Park J. et al., 2021; Cheng et al., 2021) have also been proposed that help physicians in a variety of ways. Table 4 shows the summary of healthcare knowledge graphs. The detailed and structured comparison of these KGs is also presented in the ORKG.^24^

**Table 4:** Summary of healthcare knowledge graphs.

Paper	Description	Method	Resource availability	Data used
Malik et al. (2020)	Automated KG curation framework for neurological diseases prognosis, diagnosis, and treatment	Ontology-based information extraction, ensemble learning (Random Forest, Decision Tree, Support Vector Machine, Adaptive Boosting, and Gradient Boosting) and word embedding based on skipgram	Available on Mendeley Data^a^	Electronic health records (progress notes, discharge summaries, and radiology reports) of patients with an intracranial aneurysm
Yu et al. (2017)	KG of Traditional Chinese Medicine (TCM) for interpretation, and utilization of TCM assets	Ontology-based approach, semi-automatic process (utilized text-mining tool to extract entities and relations)	-	Integrated heterogeneous data resources related to TCM
Gyrard et al. (2018)	Healthcare KG taking into account contextual and personalized knowledge about patients	Utilized different ontologies (e.g., LOV4IoT-Health, BioPortal, and Linked Open Vocabularies), rule-based reasoning	-	Integrated heterogeneous sources (sensors data, medical datasets from Alchemy API^b^, UMLS, and ICD-10^c^
Postiglione (2021)	Healthcare KG to identify possible causal relations between different healthcare entities (symptoms, diseases, and measurements) and suggest personalized treatments	Transformer language model, few-shot learning, entity linking (integrated Wikidata)	-	Data taken from cardiological departments of hospital Azienda Ospedaliera Universitaria (AOU) Federico II
Shi et al. (2017)	Developed a KG of textual medical records to support semantic querying and reasoning	Adopted a pattern-mining framework described in Nakashole et al. (2011); Ernst et al. (2015), contextual inference pruning algorithm, Naive Bayes, Logistic Regression, Support Vector Machine, and Decision Tree to classify inferences (Logistic Regression performed best)	-	Electronic health records were collected from Health Information System, Zhejiang, China
Huang et al. (2017)	KG of depression disease facilitates relevant doctors to explore various knowledge resources and to answer clinical queries	Entity and concept identification, Semantic Annotation with Xerox's NLP tool Khiari (2015)	-	Data for KG construction was taken from PubMed, ClinicalTrials.gov, DrugBank database^d^, Wikipedia Antidepressant, DrugBook, SIDER
Li et al. (2020)	Developed healthcare KG from EMRs, contains 9 types including diseases, symptoms, etc.	Vocabulary-based bidirectional maximum matching (BMM) based NER, BiLSTM-CRF model, entity recognition and normalization, relation extraction, graph embedding	Downloadable from the Appendix of paper	Medical records taken from EMR system, hospital information system, laboratory information system (LIS), and radiology information system of Southwest Hospital in China
Zhang et al. (2020)	Constructed the disease-specific, and cross lingual healthcare KG	Health Knowledge Graph Builder (HKGB), Active learning to enhance efficiency of annotations by clinicians, LSTM-CRF to extract entities, relation extraction using pattern-based method and supervised learning (Pipeline-CR-CNN Model), Entity Alignment (Dedupe-based)	-	Structured and unstructured health-related data taken from different sources
Yu et al. (2022)	Developed KGs to improve chronic disease management for children	AI Chronic Management System (AICMS), TF-IDF, and Word2Vec for text processing, classifying chronic diseases using LSTM and BERT	-	Electronic health records
Huang et al. (2019)	Children's chronic disease management KG	CRF for NER, SVM for relation recognition, and Decision Tree for entity relevance computation	-	Electronic health record (EHRs)
Cheng et al. (2021)	Developed a Knowledge Graph of Stroke	Entity extraction and linking, knowledge graph embedding	-	Harvested medical records from Xunyiwenyao.com and Dingxiangyuan

^a^ https://data.mendeley.com/datasets/gb735b78ty/1

^b^ https://bioportal.bioontology.org/ontologies/SNOMEDCT

^c^ https://bioportal.bioontology.org/ontologies/ICD10

^d^ https://www.drugbank.ca/

#### 6.1.2 Food knowledge graphs

A balanced diet is extremely crucial for a healthy life (Marx et al., 2020). Therefore, providing food information in a structured form can help individuals explore and adopt healthy dietary habits. A Food4Health knowledge graph (Fu et al., 2023) for food recommendation was developed by transforming heterogeneous datasets of food, gut microbiota, and mental disorders. The knowledge graph is useful to answer complex queries about food nutrients, the metabolism of gut microbiota and mental disorders. This KG also recommends food that has positive effect on mental disorders. Wang et al. (2022) developed a knowledge graph for food additives by obtaining the data from multiple sources. The proposed knowledge graph provides the foundation for answering questions about multiple food additives. In order to study the food safety problems the food inspection data is extracted from different web sources, and stored in the graph database to generate the knowledge graph. The KG allows visually exploring and analyzing the food safety problems according to the user requirements (Hu et al., 2022). Food knowledge graphs have also been extensively used for personalized food recommendation systems (Chen et al., 2021; Qin et al., 2020a,b; Park D. et al., 2021). Table 5 shows the summary of food knowledge graphs. The detailed and structured comparison of these KGs can be viewed in the ORKG.^25^

**Table 5:** Summary of food-related knowledge graphs.

Paper	Description	Method	Resource availability	Data source
Fu et al. (2023)	Generate food recommendations based on gut microbiota and mental health KG	Chinese Food Ontology, entity linking approach	Available online^a^	FoodData Central dataset (FDC)^b^ (McKillop et al., 2021), Integrated data about gut microbes and their metabolism^c^, NCBI taxonomy ^d^
Wang et al. (2022)	Cross-modal knowledge graph for multiple food additives	Semantic role tagging and dependency syntactic parsing	-	food inspection data
Hu et al. (2022)	KG-based approach to analyze food safety issues appeared in the food inspection data	Entity, relation and attribute extraction approaches	-	Extracted from web^e^
Chen et al. (2021)	KG-based question answering system for food recommendation	Template-based natural language question generation, Query Expansion, KG Augmentation, and Constraint Modeling	-	Dataset derives from FoodKG Haussmann et al. (2019), ADA Lifestyle Management^f^
Qin et al. (2020a)	Food spot-check KG to analyze the problems about food safety	Entities and relation extraction methods	-	Food spot-check data crawled from National Food Quality supervision and inspection center and China food and drug administration websites
Qin et al. (2020b)	KG-based question answering system for food safety	NLP based entities and relation extraction methods	-	Data was extracted from different web sources
Park D. et al. (2021)	Generate food representations and recommend food pairings using a food-chemical graph	Graph node embedding using metapath2vec	Available online^g^	recipes, chemical relations of food ingredients and chemical compounds

^a^ https://github.com/ccszbd/Food4healthKG

^b^ https://fdc.nal.usda.gov/download-datasets.html

^c^ https://www.genome.jp/kegg/compound/

^d^ https://www.ncbi.nlm.nih.gov/taxonomy

^e^ www.cfda.com.cn

^f^ https://www.tasteofhome.com/collection/dash-diet-meal-plan/

^g^ https://github.com/lamypark/FlavorGraph

#### 6.1.3 Code knowledge graphs

Numerous approaches have been proposed to extract (meta)data from software packages. Mao et al. (2019) proposed the Software Metadata Extraction Framework (SOMEF) to automate the retrieval of metadata from scientific software documentation. The framework utilizes machine learning methods to extract various types of information, such as repository name, software description, citations, and reference URLs, from README files, and then structures the data in different formats (JSON-LD, JSON, and RDF). Furthermore, the framework was extended to extract additional metadata and supplementary files (like Notebooks and Dockerfiles) from software packages (Kelley and Garijo, 2021). The extracted metadata is published in the knowledge graph to improve the search of software packages stored in repositories. Abdelaziz et al. (2020) developed a knowledge graph (CodeBreaker), and integrated in an IDE to recommend code functions while writing software. Similarly, GraphGen4Code (Abdelaziz et al., 2021) a knowledge graph was proposed that contains information about Python-based software packages hosted on the GitHub repository. The knowledge graph was created by analyzing the features of software scripts and linking them with natural language artifacts such as documentation and forum discussions obtained from StackOverflow and StackExchange. Garijo et al. (2019) presented SciSoft, a knowledge graph that contains machine-readable metadata about scientific software. The OKG-Soft framework includes: (1) an ontology to define software; (2) a method to extract and publish software metadata in a knowledge graph; (3) a platform for annotating and querying the software metadata. The benefits of OKG-Soft was demonstrated through two applications: a browser that enables users to understand environmental and social science models, and a portal that integrates climate, hydrology, agriculture, and economic software models.

Haris et al. (2022b) also proposed a method for extracting scholarly knowledge from published software packages. This method involves two steps: first, extracting software metadata by querying the Zenodo API and using SOMEF; second, using an Abstract Syntax Tree (AST)-based method to analyze the code scripts for capturing data-flow analysis. This involves analyzing Python scripts to identify input datasets, operations performed on these datasets, and output datasets. The extracted (meta)data is then published in the Open Research Knowledge Graph (ORKG). Later, this approach was extended to include software packages from various repositories (Zenodo and figshare) (Haris et al., 2023). Another significant addition was the ability to recompute code outputs. Software scripts containing scholarly knowledge were automatically executed to capture the results of operations performed on input datasets, and this detailed information was integrated into the ORKG. The resulting knowledge graph contains information about software packages, related articles, data-flow analysis information, and the results of operations. This approach simplifies the software search and enriches the ORKG with statistically significant results, which facilitates data analysis. Table 6 shows the summary of code knowledge graphs. The detailed and structured comparison of these KGs can be viewed in the ORKG.^26^

**Table 6:** Comparison of papers focusing on code knowledge graphs.

Paper	Description	Method	Data access	Scholarly knowledge	Data used
Mao et al. (2019)	Extracting metadata from software documentation	GitHub API integration, logistic regression, and multinomial naive bayes	-	×	README files taken from GitHub
Kelley and Garijo (2021)	Creating knowledge graphs of scientific software metadata	Machine learning and natural language based methods	On Github^a^ Python package^b^	×	README files
Abdelaziz et al. (2020)	Creating a knowledge graph of software scripts also focusing on data flow analysis	Used WALA library^c^ and information retrieval techniques	On Github^d^ RDF dump^e^	×	Python-based software packages taken from GitHub
Abdelaziz et al. (2021)	Generating knowledge graphs of software scripts also focusing on data flow analysis	Used WALA library and information retrieval techniques	RDF dump	×	Python-based software packages taken from GitHub
Garijo et al. (2019)	Making software metadata FAIR by publishing it in a KG	Ontology-based approach (Software Description Ontology^f^), integrated Wikidata to enrich software descriptions	Python client^g^	×	Software metadata extracted from different sources
Haris et al. (2022b)	Extracted scholarly knowledge (input datasets, operations performed on data, and output datasets) from software packages and published the extracted information into the ORKG	Abstract Syntax Tree (AST)	RDF dump, UI, SPARQL, and REST API	✓	Harvested software packages from Zenodo
Haris et al. (2023)	Extracted scholarly knowledge from software scripts with the focus on data-flow analysis as well as recomputing the results of the operations	AST, Matched code semantics in linked articles using BERT model	RDF dump, UI, SPARQL, REST API	✓	Harvested software packages from Zenodo and figshare

^a^ https://github.com/KnowledgeCaptureAndDiscovery/somef

^b^ https://pypi.org/project/somef/

^c^ https://github.com/wala/WALA

^d^ https://github.com/krassowski/jupyterlab-lsp

^e^ https://wala.github.io/graph4code/

^f^ https://w3id.org/okn/o/sd

^g^ https://github.com/mintproject/model-catalog-api

#### 6.1.4 Cybersecurity

Knowledge graph technology has also been used to present cybersecurity-related information in a structured form. A Cybersecurity Knowledge Graph (CKG) summarizes cyber event data and allows security professionals to search past cyber activities to understand future events. CKGs contain a large amount of interrelated cybersecurity information that enables cybersecurity professionals, researchers and analysts to comprehend the links between threats, vulnerabilities, attack pathways and security controls. A cyber-security KG (Deng et al., 2019) was developed to help students in searching for cybersecurity-related topics. This KG enables personalized learning and is greatly beneficial for education. Piplai et al. (2020) created a cybersecurity knowledge graph by extracting information from After-action reports (AARs). The Malware Entity Extractor (MEE) is used to extract the important entities from AARs and a neural network based model predicts the relationships between them. The CKG is augmented by merging comparable entities providing knowledge from various sources. By querying the enriched CKG, security analysts can effectively obtain results about important content published in the after-action reports. Shen et al. (2020) proposed a cybersecurity knowledge graph that combines fragmented threat data with industrial network topology to create a data-driven defense framework for industrial control network security. The authors used a distant supervised relation extraction model, ResPCNN-ATT, based on a deep residual convolutional neural network and attention mechanism to accurately extract semantic features. Different industrial control scenarios were tested using the ICSER dataset to generate a cybersecurity knowledge graph (CSKG) for analyzing the security of industrial control system.

Sarhan and Spruit (2021) proposed an Open-CyKG framework to build a Cyber Threat Intelligence (CTI) knowledge graph. It utilizes an attention-based neural Open Information Extraction (OIE) model to extract valuable cyber threat information from unstructured reports on Advanced Persistent Threats (APTs). The OIE model generates label relation triples that are further processed using the Named Entity Recognition (NER) model. Word embedding based fusion techniques are then used to organize the data and create the KG. The resulting KG allows security experts to query Open-CyKG to extract relevant information. Hooi et al. (2019) presented a framework to create threat actors knowledge graph. This knowledge graph integrates information from internet data sources and provides cybersecurity experts with detailed information to support their decision making. AttacKG was introduced by Li Z. et al. (2022) to extract structured attack behavior graphs from CTI reports and detect the associated attack techniques in an automated way. The insights gained from the threat data are used to summarize various characteristics of the techniques and expand the attack behavior graphs to technique knowledge graphs (TKGs). Table 7 shows the summary of cybersecurity knowledge graphs. The detailed and structured comparison of these KGs can be viewed in the ORKG.^27^

**Table 7:** Summary of Cybersecurity knowledge graphs.

Paper	Description	Method	Resource availability	Data source
Deng et al. (2019)	KG to browse and search cybersecurity-related concepts	word2vec, employed word embedding and similarity calculation	-	Intergrated multiple data sources (Wikipedia content and instruction materials)
Piplai et al. (2020)	Cybersecurity knowledge graph creation from malware after action reports to enable analysis of cyber-incidents	NER using CRF, Performing relation extraction using Word2vec and neural network based methods	-	Extracted AARs from various sources
Shen et al. (2020)	Cybersecurity knowledge graphs for industrial control systems	Distant supervised relation extraction model ResPCNN-ATT	Available on request	Mined threat data from multiple sources
Sarhan and Spruit (2021)	Open knowledge graph: platform for accessing and utilizing cyber threat intelligence data	Attention-based Open Information Extraction to extract relation triples from unstructured APT reports, NER	On GitHub^a^	MalwareDB Lim et al. (2017)
Hooi et al. (2019)	Creates a comprehensive knowledge graph that serves as a base for searching threat actors, including their attributes and relationships	CRF based NER	-	Data taken from the web^b^
Li Z. et al. (2022)	A technique knowledge graph from cyber threat intelligence reports to identify the adopted attack techniques	Entity extraction, co-reference resolution	On GitHub^c^	Harvested MITER ATT&CK and CTI reports from web ^d^, ^e^

^a^ https://github.com/IS5882/Open-CyKG

^b^ https://securityaffairs.co

^c^ https://github.com/li-zhenyuan/Knowledge-enhanced-Attack-Graph

^d^ https://blog.talosintelligence.com/

^e^ https://www.darpa.mil/program/transparent-computing

#### 6.1.5 Education

Chen et al. (2018b) proposed KnowEdu, a knowledge graph for education that integrates heterogeneous data (e.g., pedagogical and learning assessment data). The resulting KG contains links between pedagogical concepts and implicit pedagogical relationships. The Neural Sequence Labeling algorithm was applied to pedagogical data to extract teaching concepts, while probabilistic association rule mining was applied to learning assessment data to identify the relationships with pedagogical meaning. The applicability of the proposed approach was demonstrated by constructing a mathematics knowledge graph. Chen et al. (2018a) developed K12EduKG, knowledge graphs for K-12 education subject integration, to provide a more comprehensive and interconnected view of the various concepts and topics in the K-12 curriculum. It utilizes NER and data mining techniques to extract and link educational concepts from various sources, including textbooks and online resources. Another education KG (Dang et al., 2021) MOOC-KG (Massive Open Online Courses) was proposed to improve the use of online learning resources. The main objective was to collect and formulate the information about online courses to make them easily accessible. Similarly, another knowledge graph for MOOCs was created which integrates four major platforms: Coursera, EDX, XuetangX, and ICourse. The concepts extracted from the text attributes of the MOOCs are linked to the corresponding Wikipedia entities, resulting in a normalized representation of the knowledge and a more accurate description of the MOOCs. Li N. et al. (2022) proposed a deep learning-based method (BiLSTM-CRF) to construct knowledge graphs that integrate multimodal teaching tools. The applicability of the proposed method was demonstrated using an example of data structure course. Similarly, other knowledge graphs for education domain have also been proposed, including CKGG (Shen et al., 2021) (Chinese Knowledge Graph for High-School Geography Education), knowledge graph for entrepreneurship publication management (Chi et al., 2018), college curriculum recommendation knowledge graph (Hubert et al., 2022), and knowledge graphs for smart education (Meissner and Köbis, 2020; Sun and Gu, 2021). Table 8 presents the summary of education knowledge graphs. The overview of these approaches is also presented in structured form in the ORKG.^28^

**Table 8:** Summary of education knowledge graphs.

Paper	Description	Method	Data Availability	Data used
Chen et al. (2018b)	KG for education domain containing instructional concepts and implicit educational relations	CRF and neural sequence labeling	-	Heterogeneous data (pedagogical data and learning assessment data)
Chen et al. (2018a)	Education KG which integrates educational data like curriculum standards and prerequisite relations between educational concepts	NER using CRF, identified relations using probabilistic association rule mining	-	Chinese curriculum standards of mathematics
Dang et al. (2021)	KG of massive open online courses to use educational resources efficiently	Data mining methods and entity disambiguation,	-	Taken courses data from Coursera, EDX, XuetangX, and ICourse
Li N. et al. (2022)	Constructed multi-modal educational knowledge graphs that integrate teaching concepts, educational relationships as well as embedded teacher audio into the knowledge graph	BERT model, BiLSTM-CRF	-	Course outline, Baidu entries, Jianshu articles, offline classroom data (courseware, textbooks, and teacher audio)
Shen et al. (2021)	Constructed a Chinese KG for the geography domain for high-school geography education	CKGG ontology ^a^, BERT-based model	On GitHub^b^	Harvested geographical data from different data sources
Chi et al. (2018)	KG-based model to manage scholarly articles and combines scientific entities and concepts	Entity extraction using data mining techniques	-	Metadata extracted from articles taken from Web of Science, Engineering Village, and EBSCO
Hubert et al. (2022)	KG-based university curriculum recommendation	EducOnto	Publicly available^c^	Data for KG were collected through an online survey
Meissner and Köbis (2020)	Creating educational knowledge graphs for higher education. The approach was showcased on a reading assignment in an educational science class	Used T-Mitocar (TM) to generate association nets from texts, converted data into the RDF format Tarql^d^ and custom mappings^e^	-	Educational science data
Sun and Gu (2021)	Integration of teaching experience and domain knowledge for AI-assisted education	Information extraction, relationship mining, digitalized apprenticeship, entity recognition, and fusion of fuzzy knowledge	-	Web of Science journal literature from 2009 to 2019

^a^ purl.org/edukg

^b^ https://w3id.org/ckgg/1.0/ontology/

^c^ https://github.com/nju-websoft/CKGG

^d^ https://tarql.github.io/

^e^ https://gitlab.com/Tech4Comp/t-mitocar-rdf-transformation

#### 6.1.6 Geoscience

Wu et al. (2022) proposed a climate knowledge graph to help researchers obtain information about climate data for analysis purposes. To build the knowledge graph, data was taken from atmospheric climate summaries, OpenStreetMap and Wikidata. The Linkclimate platform is designed to enable users to search, discover and access climate data from different sources via a unified interface. It also provides tools for data analysis and visualization that can help researchers to better understand complex climate systems. Deng et al. (2021) proposed a large-scale multimodal KG based on 1.12 million scholarly articles. In addition to the metadata information, tables, text information of the articles, knowledge entities, the temporal and spatial attributes of the articles were extracted to combine multimodal scholarly data and features. In particular, the proposed approach aims to extract geoscience knowledge entities using machine learning and information retrieval techniques while keeping geoscientists in the loop. Zhou et al. (2021) proposed geoscience knowledge graph consists of a complex model (entity representation model) to represent spatio-temporal information as well as relationship between geo-objects and entity objects. The federated crowd- intelligence collaboration method and the dynamic abstraction method were used to build the geoscience knowledge graph. The applicability of the KG was demonstrated using three applications, from inference to the construction of high-resolution geological time scales and intelligent mapping. Tang et al. (2022) proposed a multidimensional method for the development of petro-onto and petro-KG for the multi-stage business chain of enterprises in the petroleum field. The entities, attributes and relationships of Petro-KG are established by collecting Sinopec's multi-source and multi-structured data. Table 9 shows the summary of geoscience knowledge graphs. The detailed and structured comparison of these KGs can be viewed in the ORKG.^29^

**Table 9:** Comparison of Geoscience knowledge graphs.

Paper	Description	Method	Data availability	Data used
Wu et al. (2022)	Development of an interoperable platform for constructing and using knowledge graphs for climate data	Entity linking, ontology alignment	On GitHub^a^, SPARQL endpoint^b^	Data taken from OpenStreetMap, Wikidata, the national oceanic and atmospheric administration's daily climate summaries
Deng et al. (2021)	Development of a multimodal knowledge graph for geoscience academic research	Human-In-the-Loop framework, link prediction	Available online^c^, SPARQL endpoint^d^	Scholarly articles
Zhou et al. (2021)	Construction and applications of a knowledge graph for managing and analyzing geoscience data	Entity object representation model, federated crowd intelligence collaboration, entity embedding model, entity link prediction model	-	Heterogeneous genoscience data (complex spatiotemporal information and text)
Tang et al. (2022)	Multi-sourced KG for managing and utilizing information related to petroleum exploration and development	Conditional random field for entity recognition, attribute extraction using rule-based methods relation, attribute extraction	-	Sinopec's multisourced heterogeneous data

^a^ https://github.com/futaoo/LinkClimate

^b^ http://jresearch.ucd.ie/kg/

^c^ https://gakg.acemap.info/

^d^ https://snorql.acemap.cn/

#### 6.1.7 Engineering

Knowledge graph technology has also been used for describing the important information of engineering domain in structured form (Fan et al., 2021). Yan et al. (2020) constructed a knowledge graph for smart manufacturing equipment such as lathes, conveyors and robots. First, the heterogeneous data is collected from the network of devices. Second, using the conditional random fields (CRF) algorithm, the device-related information (device name, product location, and company name) is extracted and then the relationships between the different device entities are determined. Third: presented the extraction knowledge into the KG. The relevant communities can utilize this KG to extract information about different devices during manufacturing. Fan et al. (2020) proposed a knowledge graph for power grids using two-fold approach. First, a corpus was created using the power dispatching behaviors. Second, a BiLSTM-CRF model was developed to extract entities and identify the relationship patterns of dispatching behavior. Such a KG can be helpful in searching the knowledge related to the power dispatching behavior and the dispatching automation system. Yang et al. (2019) developed a knowledge graph for the energy domain by integrating multiple heterogeneous data sources. The KG provides basis for mining and visualizing important information about the power domain. Stewart et al. (2024) proposed a maintenance knowledge graph for maintenance that utilizes maintenance work orders (MWO) data. The KG captures important information about industrial assets and was developed using two interconnected software systems—Echidna and MWO2KG. Echidna is an interface for visualizing and querying maintenance orders, while MWO2KG is a method for creating a knowledge graph. These two tools offer the possibility to query and analyze huge amounts of historical assets data efficiently.

Jiang et al. (2021) proposed a knowledge graph for systematic knowledge management of Construction Safety Standards (CSS). The developed KG facilitates the analysis, retrieval and sharing of knowledge about safety standards. Zhao et al. (2018) constructed the knowledge graph for the aviation risk domain. Creating the knowledge graph of the aviation risk domain effectively improves the efficiency of knowledge retrieval and supports decision making by analyzing historical data. Zhang et al. (2019) proposed a knowledge graph of Maritime Dangerous Goods (MDG) to share, disseminate and utilize the knowledge of these goods to support intelligent transportation. Meng et al. (2022) proposed a knowledge graph for incorporating data about electric equipment faults which facilitates automatic fault diagnosis by means of questioning answering. Liu et al. (2021) proposed a knowledge graph of railway incidents. The KG was generated by systematically analyzing the reports mentioning railway incidents. The results show that such a KG can help railway operators to retrieve and understand past accidents easily, thus helps to make accident prevention decisions. Several other knowledge graphs structure content across various engineering domains including, spacecraft launch (Chao et al., 2021), building automation systems (Dibowski and Massa Gray, 2020), urban planing (Grisiute et al., 2021), manufacturing engineering (He and Jiang, 2019; Buchgeher et al., 2021), and civil/pavement engineering (Yang et al., 2022). Table 10 presents the summary of engineering domain knowledge graphs. A detailed and structured comparison of these KGs is available in the ORKG.^30^

**Table 10:** Comparison of engineering knowledge graphs.

Paper	Description	Method	Data availability	Data used
Yan et al. (2020)	Manufacturing equipment KG, providing means for intelligent equipment information retrieval and utilization	Data mining and integration, regular expression filtering, entity extraction using CRF	-	Manufacturer's product information data and teaching laboratory equipment records
Fan et al. (2020)	KG of a power dispatching data, identifying dispatching behavior relationship patterns	Annotated data using entropy-based phrase extraction method, entity extraction using BILSTM-CRF model	-	Retrieved data related to power dispatching behavior from different sources
Yang et al. (2019)	KG for power assets	Ontology construction, Entity Alignment Matching, Similarity-based Matching	-	Electric Power Knowledge Base Manual Test Records Power Information Systems
Stewart et al. (2024)	Visualizing, querying, and identifying failure modes through maintenance data KG	Performed NER using Flair ^a^	Available on GitHub ^b^	Maintenance work orders data
Jiang et al. (2021)	Management of construction safety standards using KG	Knowledge structure analysis, entity extraction	-	Construction safety standards data
Zhao et al. (2018)	Organization of the cases of aviation risk events	Aviation risk event ontology, pattern-based knowledge mapping, R2RML mapping	-	American Aviation Safety Reporting System (ASRS)
Meng et al. (2022)	Creating a KG of electric power equipment faults to facilitate the automatic fault diagnosis	extracts electric power equipment entities using BERT–BiLSTM–CRF	-	Chinese technical literature
Liu et al. (2021)	Facilitating exploration of railway operational accidents using KG	Topological analysis method	Data taken from RAIB^c^	Rail accident investigation reports
Dibowski and Massa Gray (2020)	Computerized engineering of building automation Systems using a KG	Brick and QUDT ontologies, Semantic tagging, describing functional and non-functional requirements for the building automation Systems in the KG	-	Data from Building Information Modeling (BIM) files
He and Jiang (2019)	Developed manufacturing KG to answer production-related problems	Ontology-based MKG construction with unified MK-filter, Graph-oriented meta-knowledge model to represent entities	-	Manufacturing knowledge taken from production practices, and different web platforms
Yang et al. (2022)	Integrated and analyzed knowledge assets of pavement information for intelligent decision-making	BiLSTM-CRF based NER	-	pavement inspection report text taken from different institutions ^d^

^a^ https://github.com/flairNLP/flair

^b^ https://github.com/nlp-tlp/mwo2kg-and-echidna

^c^ https://www.gov.uk/raib-reports

^d^The Research Institute of Highway Ministry of Transport, National Center for Quality Supervision.

#### 6.1.8 Datasets knowledge graphs

Färber and Lamprecht have developed a knowledge graph for datasets called DSKG (Faerber and Lamprecht, 2021), which aggregates metadata from different datasets published in different research areas. For the construction of the knowledge graph, only the datasets whose metadata are available in the scholarly articles were considered. To address the problem of ambiguity in author names, a novel method for disambiguating author names was introduced to ensure that each author in the knowledge graph has a unique identifier. The knowledge graph is further enhanced by integrating links to ORCID, Wikidata and Microsoft Academic Knowledge Graph (MAKG), which facilitates its connection to the Linked Open Data Cloud. The DSKG serves as a foundation for the development of various applications, such as dataset search, scholarly data analysis and dataset impact assessment. It also supports machine learning applications through the use of embeddings created with RDF2Vec. Brickley et al. (2019) have developed a dataset discovery tool that provides search functionality for all datasets available on the Web. In addition, the proposed KG operates within an open ecosystem in which dataset owners and providers publish metadata with semantic representations on their websites. This metadata is then aggregated, normalized and reconciled using the proposed approach to facilitate the discovery of the published datasets. Ojo and Sennaike (2019) constructed knowledge graphs from open data catalogs to uncover latent relationships and the value of datasets based on sociometric profiles. The proposed method consists of two main steps: first, the relatedness of datasets is computed using a self-organizing map (SOM), and second, the SOM is transformed into a knowledge graph through topological distances between the datasets. The developed knowledge graphs facilitate the identification of dataset clusters and topics, enable the discovery of important datasets and improve recommendations for related datasets.

Younsi Dahbi et al. (2020) presented the KG4OGD, a knowledge graph that aims to bring open government data into a structured form to gain valuable insights. The authors have proposed a comprehensive knowledge representation model to support the construction of KG4OGD that focuses on both metadata and content. This model was developed to enrich the descriptive and contextual background of the data to improve the coherence and consistency of the knowledge graph. The proposed model was specifically applied to the public health sector data, which has gained much importance due to the global pandemic context. Wang et al. (2017) worked on a pilot project that aimed to integrate Research Graph datasets into external web services via JSON-LD, improving access to research datasets, funding data, publications and researcher profiles. The project tests the conversion of Research Graph Registry objects to JSON-LD using vocabularies from Schema.org and includes examples from well-known research institutions. It also makes these datasets semantically available as linked data and discusses possible extensions of the Schema.org vocabulary for scholarly communication. Table 11 shows the summary of datasets knowledge graphs. The detailed comparison of these knowledge graphs can be viewed in the ORKG.^31^

**Table 11:** Comparison of datasets knowledge graphs.

Paper	Description	Method	Data availability	Data used
Faerber and Lamprecht (2021)	A framework for making datasets widely accessible as linked open data source	Data transformation and integrating, utilizing DCAT vocabulary, Utilizing ORCID records for author name disambiguation	Publicly available ^a^,^b^ Färber and Lamprecht (2021)	Retrieved datasets information from OpenAIRE, Wikidata and MAKG
Brickley et al. (2019)	Constructed a KG that supports searching datasets available on the Web	Retrieves the metadata from the Web, Data cleaning and normalization, Indexing and ranking of results	Publicly available ^c^	Mined different web sources
Ojo and Sennaike (2019)	Knowledge graph of open data catalogs to improve discovery and recommendation of related datasets	Self-organizing Maps (unsupervised artificial neural network)	-	DubLinked
Younsi Dahbi et al. (2020)	Transforming healthcare data into a KG to gain valuable insights	Knowledge representation model for enhancing descriptive and contextual metadata, GovDataset Ontology	-	Open government raw data
Wang et al. (2017)	Integrating research graph records to enhance accessibility and interoperability of data as well as support linked data	Research Data Switchboard open source software	-	Research datasets, funding records, publications and researcher records

^a^ http://dskg.org

^b^ https://github.com/michaelfaerber/data-set-knowledge-graph

^c^ https://datasetsearch.research.google.com/

### 6.2 Domain-agnostic knowledge graphs

DBpedia (Auer et al., 2007), Google Knowledge Graph, Microsoft Concept Graph (Ji et al., 2019), Wikidata, SemOpenAlex, OpenAIRE and Open Research Knowlege Graph (ORKG) are general-purpose knowledge graphs. These KGs, except ORKG provide rich metadata about articles, researchers, organizations, and projects. However, their focus is not exclusively on structuring scholarly knowledge.

Open Research Knowledge Graph (ORKG), on the other hand, is an infrastructure designed specifically for representing the scholarly knowledge expressed in research articles. It aims to integrate, represent, and disseminate research findings in a structured and semantically rich manner, making it unique among the listed knowledge graphs for its exclusive focus on scholarly knowledge. ORKG supports describing the essential information of scholarly articles in the form of research contributions—the core element of information in the ORKG.

These structured descriptions are guided by ORKG templates^32^ i.e., graph patterns that specify properties and values. Templates can be used to create ORKG content, in particular to describe research contributions. Templates not only facilitate the structuring of scholarly knowledge but also enable the linking of used terms (classes and properties) to external semantic resources (ontologies), thereby ensuring interoperability and reusability of content created in ORKG. These resources can be linked through data exchange methods: REST API and SPARQL. ORKG provides runtime support to identify and integrate semantic resources while curating the content.

If various studies reference the confidence interval in their experimental measurements, a corresponding template can refer to the Statistical Methods Ontology (STATO). When a paper contribution description employs this template to define properties with dynamic values, these properties are automatically linked to the ontology. This process involves the creation of a confidence interval class and the specification of constraints for template properties. The EMBL-EBI Ontology Lookup Service (Côté et al., 2010) (OLS) and its REST API are used to facilitate the lookup of semantic resource classes served by the OLS, allowing for the definition of value ranges in the templates.

Knowledge graphs provide added value beyond traditional metadata systems by enabling fine-grained semantic representation of research content. For example, instead of linking only a publication to a dataset, a knowledge graph can represent relationships between specific entities such as methods, variables, and results, allowing users to query questions such as “which diagnostic method achieves the highest accuracy for a given condition.” Similarly, knowledge graphs support semantic reasoning, enabling the discovery of implicit relationships (e.g., identifying related research problems across domains). Furthermore, they facilitate structured comparison of research contributions, as demonstrated in ORKG, where multiple papers can be compared along shared dimensions such as methods, datasets, and evaluation metrics.

## 7 Interoperable research information systems

This section synthesizes the findings of Sections 3–6 by focusing on the functional interrelationships between the reviewed systems. Rather than treating scholarly identifier systems, bibliographic databases, research data management services, and knowledge graphs as isolated components, we analyze how they jointly form an interoperable research information ecosystem that underpins scholarly communication. Figure 2 illustrates the interactions among these systems and highlights how their interoperability facilitates seamless access, management, and dissemination of research artifacts. In the following, we provide concrete examples to illustrate these interdependencies in practice.

### 7.1 Persistent identifiers and scholarly identifier systems

In Section 3, Scholarly identifier systems are consistently described as the foundational referencing layer that enables interoperability across research information systems. Persistent identifiers (PIDs) such as DOIs, ORCIDs, and ROR are crucial for uniquely identifying a wide range of scholarly artifacts including research papers, datasets, books, researchers, and organizations. Persistent identifiers (PIDs) are integral to research data management (Demchenko et al., 2024), as they provide stable and non-volatile references to datasets, software, and other research outputs published through RDM services. For example, when a dataset is published in a RDM service like figshare or zenodo, it is typically assigned a DOI to ensure it can be consistently cited and linked with other research artifacts, regardless of its physical location.

PIDs are also important for the *Bibliographic databases* as they ensure that references and citations in bibliographic databases like PubMed or Crossref are accurate and traceable. They allow these systems to reliably link citations to the exact research outputs. Several knowledge graphs and scholarly identifier systems integrate other scholarly identifier systems to produce the complete life cycle of publishing artifacts and attributing them on the respective profiles of authors (Haak et al., 2012). For example, the ORKG integrates DataCite to persistently identify the machine-readable artifacts, whereas DataCite and ORCID interoperates to retrieve and attribute the research artifacts. ORKG allows users to persistently identify their papers and corresponding comparisons by leveraging DataCite services (Haris et al., 2021, 2022a). The metadata of these artifacts including ORCID is shared with DataCite during publishing the artifacts. In general, there exists interoperability between DataCite, ORCID, and OpenAIRE that enables the exchange of metadata and the attribution of research artifacts across infrastructures (Cousijn et al., 2021). When research outputs are registered with DataCite using associated ORCID identifiers, the corresponding records are propagated to authors' ORCID profiles and also indexed by OpenAIRE, thereby supporting integrated discovery and attribution workflows across research information systems.

### 7.2 Bibliographic databases

Section 4 shows that bibliographic databases function as aggregation and discovery systems that collect, index, and interlink scholarly metadata at scale. These systems index information about scholarly publications and often serve as the backbone for academic reference management and literature search. Bibliographic information from these systems is commonly used to populate knowledge bases with detailed metadata about publications. Such rich metadata also enables enhanced semantic queries at the granular level. For example, Sadeghi et al. (2017) developed a knowledge graph of scholarly communication metadata by integrating DBLP and the Microsoft Academic Knowledge Graph, thereby enabling efficient query processing over a unified knowledge base. Bibliographic databases further rely on persistent identifiers to resolve and retrieve detailed publication records, ensuring that references within scholarly articles are accurate and verifiable.

### 7.3 Research data management services

As discussed in Section 5, Research Data Management (RDM) services support the organization, storage, preservation, and sharing of research data. Artifacts published via RDM services are often exported to Bibliographic databases to enhance the discoverability of these artifacts. This metadata includes details including authors, title, publication date, and the DOI of related research artifacts. RDM services also interoperate with publishers. A notable collaboration between Elsevier and PANGAEA involves the cross-linking of content related to earth systems research. PANGAEA automatically connects its research datasets with related articles in Elsevier journals on ScienceDirect, and vice versa, thus facilitating data flow into reliable archives and enhancing the verification and use of the related scientific data.

### 7.4 Knowledge graphs

Knowledge graphs act as a unifying semantic framework that links identifiers, publications, datasets, and domain knowledge across heterogeneous sources. By leveraging persistent identifiers, knowledge graphs can accurately connect research artifacts originating from RDM services, bibliographic databases, and publishing platforms, thus ensuring consistent attribution and traceability across the graph. While RDM services focus on the curation, preservation, and dissemination of individual research artifacts, knowledge graphs complement these services by integrating such artifacts into a unified semantic structure. Through the combination of structured and unstructured data sourced from RDM platforms, knowledge graphs enable advanced querying, contextualization, and cross-domain exploration of research outputs, thereby extending the value of curated research data beyond their original repositories.

### 7.5 Cross-knowledge-graph alignment

In addition to integrating data within a single system, interoperability increasingly requires alignment across multiple knowledge graphs. This is typically achieved through shared ontologies, schema mappings, and the use of persistent identifiers to link equivalent entities across graphs. Federated querying approaches further enable users to access information from multiple knowledge graphs without requiring full data integration. Such alignment supports more comprehensive knowledge discovery, but also introduces challenges related to semantic heterogeneity, schema differences, and varying data quality across sources.

### 7.6 End-to-end workflow examples

A researcher deposits a dataset in Zenodo, where it is assigned a DOI via DataCite. The metadata record is enriched with the depositor's ORCID identifier and the funding organization's ROR ID. Through interoperability between DataCite and OpenAIRE, this metadata is harvested and indexed within the OpenAIRE Research Graph. Data published through RDM platforms is often integrated into knowledge graphs (Haris et al., 2023, 2024; Mao et al., 2019), where it forms the basis for advanced analysis and visualization.

Similarly, in domain-specific scenarios such as earth sciences, datasets published in repositories like PANGAEA are linked to publications via persistent identifiers. These links are aggregated by bibliographic datasets such as OpenAIRE and integrated into knowledge graphs, where they support semantic querying, cross-dataset analysis, and domain-specific applications.

Within knowledge graphs, semantic links between datasets and related publications are established based on shared research problems and concepts. In this way, data originating from RDM platforms becomes part of a structured knowledge graph that supports advanced analysis and cross-artifact discovery. Researchers can then query the knowledge graph to retrieve information about related artifacts, including datasets, software, and publications.

Research articles published with DOIs are indexed in bibliographic databases such as Crossref. Through DataCite and Scholix, links between publications and their underlying datasets are established. Knowledge graphs extract structured information from publications, such as methods, datasets, and results, using information extraction or manual curation, and represent them as interconnected entities. This enables advanced use cases such as question answering, cross-paper comparison, and recommendation.

### 7.7 Integration mechanisms and design considerations

The interoperability described above is enabled through a range of standardized integration mechanisms, including REST APIs, OAI-PMH harvesting, SPARQL, and GraphQL-based endpoints. These protocols support data exchange, querying, and integration across distributed systems. However, integrating heterogeneous sources also introduces challenges related to provenance tracking, versioning of metadata, entity reconciliation, and conflict resolution. For example, the same research artifact or any other research object may appear in multiple systems with slightly differing metadata that requires reconciliation strategies based on persistent identifiers and similarity matching. Addressing these challenges is essential for ensuring consistency, trustworthiness, and reproducibility in interconnected research information systems.

## 8 Discussion

We have presented various Research Information Systems, including persistent identifier (PID) systems, bibliographic databases, Research Data Management (RDM) services, and knowledge graphs, and discussed their potential benefits. Additionally, we have curated a wide range of articles from diverse disciplines, in a structured manner within the Open Research Knowledge Graph (ORKG) to facilitate the efficient utilization of vital information published in these articles. These detailed descriptions are then compared in tabular form using the ORKG services. Such comparisons enable us to conduct a variety of experiments, for instance, addressing the quantitative questions outlined in Section 1. In this section, we show the usage of ORKG data to answer these questions as well as provide the summary of research information systems.

### 8.1 Analyzing ORKG structured data

**Figure 4:**
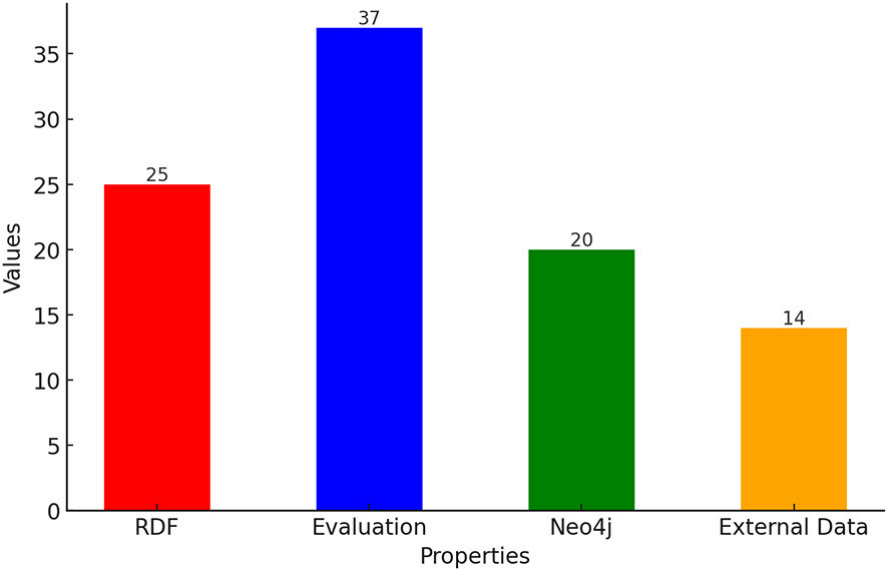
The bar chart shows the frequency of available properties described in reviewed articles. It also shows the number of articles that contain details about the underlying storage mechanism: RDF or Neo4j database.

To answer the questions described in Section 1, we have analyzed the content of these comparisons focusing on specific attributes. Figure 4 presents answers to several questions. It shows the number of articles that report on the evaluation of results and provide details about the data used. Additionally, it shows the number of articles that provide information on the methods used to store the extracted data. Note that these results have been generated based on the data available in the ORKG. By using the structured descriptions, we can also answer complex queries, such as retrieving all papers focusing on healthcare and COVID-19 knowledge graphs, and using PubMed datasets and BERT-based methods to extract important entities from documents. Another example of a (meta)data analysis performed using the comparison of COVID-19 Reproductive Number Estimates can be found in Oelen et al. (2020a).

The ORKG enhances the global discovery of its research artifacts through the assignment of persistent identifiers. By publishing all comparisons with DOIs, we ensure their discovery in various information systems, including DataCite, OpenAIRE, and ORCID. During the publication process, DOIs of the referenced articles is included in the metadata of each comparison to enable a seamless link between the articles and the associated comparisons. This approach of linking through metadata facilitates the discovery of comparisons in global information systems. As a result, users exploring research articles can discover these artifacts, allowing them to gain an overview of the current state of the art. Our aim is to continuously update these comparisons to provide an up-to-date, structured and concise overview of the state of the art.

### 8.2 Persistent identifiers

Persistent Identifiers (PIDs) are essential for scholarly communication infrastructures. They provide a persistent, unique, and machine-readable identifier for research artifacts. Assigning PIDs to research artifacts ensure discoverability and accessibility, which can ultimately increase the visibility and impact of scholarly work. PIDs also facilitate interoperability among research infrastructures which potentially enable accessing research artifacts in a federated manner.

### 8.3 Bibliographic databases

Bibliographic databases encompass metadata about research artifacts (research articles, datasets, software, instruments etc.) and other entities authors, projects, and organizations. This review highlights the importance of bibliographic databases in facilitating literature search and other entities, and research impact assessment. These infrastructures have the potential to improve scholarly communication by opening new ways to navigate and explore the research artifacts and other objects.

### 8.4 Knowledge graphs

The advancement of knowledge graphs holds significant promise for enhancing the accessibility and reusability of research artifacts. They offer variety of ways for searching, accessing and using scholarly knowledge, thereby facilitating important discoveries and insights across diverse disciplines. One of the key advantages of knowledge graphs lies in their ability to represent and manage structured and formally represented knowledge at different formalization levels, including unstructured and semi-structured information, as well as assertional and taxonomic information. This integration streamlines the data connectivity, discovery as well as enables the development of various applications, including intelligent question-answering systems and personalized recommendation engines.

We have also presented the interoperability mechanism between these systems and also showed with examples that how these systems together form a tightly coupled research information ecosystem. Scholarly identifier systems provide the stable referencing backbone that enables consistent linking across bibliographic databases, research data management services, and knowledge graphs. Bibliographic databases aggregate and curate scholarly metadata, which in turn informs both RDM workflows and knowledge graph construction. RDM services act as authoritative sources of curated and persistently identified research artifacts, while knowledge graphs integrate and contextualize information originating from all three subsystems. Most importantly, these relationships are mutually reinforcing: insights derived from knowledge graphs can inform metadata enrichment in bibliographic databases and support improved data organization and reuse in RDM services, thereby closing the loop within research information systems.

### 8.5 Future directions

We have emphasized the importance of various research information systems for sharing and usability of research artifacts. To further improve the accessibility and usability of artifacts, we suggest the following future directions:

#### 8.5.1 Data availability

The availability of data plays a crucial role in research. Throughout the research lifecycle, enormous data is generated that need to be available on RDM services to enable further research. While numerous knowledge graphs have been developed, the materials used to generate the results often remain inaccessible, which hinders reproducibility. The practice of publishing supplementary material is not yet widespread among researchers. It is important to embrace RDM practices to disseminate research data effectively, thereby enhancing its usability.

The metadata-based linking of research artifacts is interesting in order to establish meaningful relationships between them. When publishing content on data management services (e.g. Zenodo, figshare, etc.), links to supplementary material must be provided. If researchers provide these links at the time of publishing their data, the linked artifacts can be easily discovered with machine-readable links already defined, thereby improving the contextual understanding of a research work. However, such a practice is not widespread among researchers. This oversight can lead to fragmented data, with valuable artifacts remaining isolated and underutilized. If researchers consistently incorporate metadata-based links during the data publication process, the resulting artifacts become more discoverable through machine-readable links, thus improves the discoverability of underpinned artifacts and other research results as well as enables automated systems to recognize relationships between different research artifacts.

#### 8.5.2 Data exploration

Exploring knowledge graphs through a user interface is an interesting aspect for research communities. A well-designed user interface can significantly enhance the accessibility and usability of knowledge graphs for users. Knowledge graphs need to be equipped with user-friendly interfaces that allow researchers to navigate through the data effectively. Such interfaces should provide advanced query capabilities that allow users to search and explore research artifacts and gain detailed insights.

#### 8.5.3 Knowledge graphs population

The constructed knowledge graphs suffer from data incompleteness. Focusing on completing these KGs will provide contextual information. Since ORKG supports the description of research work presented in articles in a structured form, population, completion, and accuracy of its data are crucial for its widespread usage. Publishing research artifacts by following Findable, Accessible, Interoperable, and Reusable (FAIR) data principles is important for their discovery and usability.

Currently, natural language processing (NLP) methods are widely used to extract specific entities from text data. These methods are trained on vast text corpora and can identify and categorize important entities from documents. However, the accuracy of these NLP techniques is arguable, especially when it comes to the granularity required, such as capturing data-flow analysis (input data, methods applied to it and output data).

The current limitations of NLP techniques in creating knowledge graphs highlight the need to explore alternative approaches such as static code analysis and other reliable programmatic methods. Static code analysis is considered as an alternative route for extracting structured scholarly knowledge from published software packages and should be widely used. The scholarly knowledge published in research articles is conceptualized in the corresponding software packages, and this knowledge can be extracted using the code analysis methods as explained by Haris et al. (2022b, 2023). This approach provides a less resource-intensive way to extract structured scholarly knowledge at the required granularity. However, the presented approaches focus only on the analysis of Python scripts and Jupyter Notebooks. The code analysis method can be extended to scripts written in other languages (R, Java, MATLAB) to extract code semantics from them—potentially enabling the extraction of rich descriptions in a structured manner.

Many data sources, including Zenodo, PANGAEA, figshare, and dryad, comprise an extensive collection of datasets across various domains. A promising avenue for future exploration could be to analyze these datasets and related scientific articles to extract important information. The most valuable insights from vast amounts of research data and publications can be extracted using advanced data extraction techniques such as LLMs. Publishing such information in the ORKG will potentially populate this infrastructure with rich machine-readable descriptions of articles. This approach enhances the accessibility and discoverability of research results, as well as facilitates the integration and synthesis of knowledge across different scientific domains.

#### 8.5.4 Development of advanced applications

To significantly enhance the functionality of research information systems, the development of specialized downstream applications is crucial. These systems offer a solid ground for creating tools that streamline and innovate various facets of the research process, thereby increasing productivity across different research domains.

One important application is the automation of literature reviews. Tools designed for this purpose support the machine-assisted collection, analysis, and summarization of relevant literature based on predefined topics or queries. This not only accelerates the research phase but also ensures comprehensiveness, and helps researchers in maintaining an up-to-date understanding of their field.

Recommender systems have gained quite a lot traction in both research and industry. KG technology has been widely adopted to develop advanced recommender systems (Guo et al., 2022). A software recommender system can be developed to suggest relevant software to users based on their queries. GitHub contains plethora of software and filtering them manually according to specific criteria is both time-consuming and cumbersome. By developing a system that analyzes the semantic details of software packages, it becomes possible to suggest the most relevant software that closely aligns with user queries.

Advanced search capabilities that utilize semantic search, natural language processing, and machine learning algorithms are also an important area of research. These capabilities enable researchers to uncover relevant studies that might be buried under the sheer volume of scholarly articles, which is particularly beneficial for those investigating niche or rapidly evolving topics.

Federated data retrieval is another important aspect of search. Federated data retrieval meets the needs of data-driven applications by providing an efficient method for accessing and analyzing data from distributed sources. These systems enable scalable solutions by leveraging distributed data sources, reducing the need for centralized processing. Various services have been developed to retrieve data in a federated manner. However, as mentioned above, the availability of these services is essential to enable advanced applications. The filtering of query results at the metadata level is widely observed, but filtering at the content level has not gained much attention. Haris et al. (2021) developed a GraphQL-based federated query service that integrates multiple research information systems along with the ORKG, allowing researchers to perform complex queries and filter the results at both metadata and content levels. This approach enables complex (meta)data-driven analysis. Such services need to be developed on a broader scale to retrieve the data from disparate sources and provide more comprehensive search and analysis tools to different stakeholder groups.

## 9 Conclusion

The development of research information systems has revolutionized scholarly communication. These systems have opened up new possibilities for sharing and improving the usability of research artifacts. In addition, the structured representation of research artifacts in the form of knowledge graphs has the potential to lead to new applications and scientific advances. We have discussed the current state of various research information systems and highlighted their role in the sharing and reuse of research artifacts. We have also provided detailed information about these systems in a format that is both comparable and machine-readable. This approach facilitates efficient analysis and reuse of the information contained in the corresponding articles. For better discovery and management of scholarly knowledge, the integration of bibliographic databases, research data management services and knowledge graphs is not only beneficial but essential. By focusing on better interconnected and accessible research information systems, researchers can easily discover and leverage vast amounts of data and gain new insights for further research. The ultimate goal should be to create more open, accessible and efficient networks of research information systems to support the easy sharing, access and reuse of scholarly knowledge, thereby enhancing innovation and enabling the development of advanced applications.

## Footnotes

1
https://datacite.org/

2
https://api.datacite.org/graphql

3
https://www.semanticscholar.org/

4
https://www.wikidata.org/w/api.php

5
https://unpaywall.org/

6
https://opencitations.net/

7
https://intelcomp.eu/

8
https://oipub.com/

9
https://www.linknovate.com/

10
https://commons.datacite.org/

11
http://wikicite.org/

12
https://zenodo.org/

13
https://www.pangaea.de/

14
https://figshare.com/

15
https://datadryad.org/stash

16
https://dataverse.org/

17
https://data.mendeley.com/

18
https://vivli.org/

19
https://ckan.org/

20
https://www.ala.org.au/

21
https://www.earthchem.org/

22
https://www.pangaea.de/about/services.php

23
https://www.w3.org/TR/rdf-concepts/

24
https://doi.org/10.48366/R677634

25
https://doi.org/10.48366/r589671

26
https://doi.org/10.48366/r589614

27
https://doi.org/10.48366/r597897

28
https://doi.org/10.48366/R677583

29
https://doi.org/10.48366/r597878

30
https://doi.org/10.48366/r598216

31
https://doi.org/10.48366/R677334

32
https://orkg.org/templates
